# Dextran Sulfate Effects EMT of Human Gastric Cancer Cells by Reducing HIF-1α/ TGF-β

**DOI:** 10.7150/jca.55550

**Published:** 2021-04-12

**Authors:** Yun-ning Huang, Yuan-yi Xu, Qian Ma, Meng-qi Li, Jia-xin Guo, Xiaofei Wang, Xiu Jin, Jing Shang, Long-xing Jiao

**Affiliations:** 1 Department of Gastrointestinal Surgery, The Affiliated People's Hospital of Ningxia Medical University, Yinchuan, Ningxia , P.R. China.; 2 Department of Pathology, Ningxia Medical University, Yinchuan, Ningxia, P.R. China.; 3 College of Life Sciences, Ningxia University, Yinchuan , Ningxia, P.R. China.; 4 College of Basic Medicine, Ningxia Medical University, Yinchuan, Ningxia, P.R. China.; 5 Department of Pathology,North China University of Science and Technology Affiliated Hospital, Tangshan, Hebei, P.R. China.; 6 Department of Pathology, Affiliated Hospital of Jining Medical University, Jining, Shandong, P.R. China.

**Keywords:** Dextran sulfate, Human gastric cancer, HIF-1α, EMT.

## Abstract

The peritoneal implant metastasis is one of the main pathway and main cause for high mortality for gastric cancer metastasis. Researchs show that epithelial-mesenchymal transition (EMT) playing essential role in modulating gastric cancer metastasis, and the expression of hypoxia inducible factor-1α (HIF-1α) can promote EMT in tumor cells. This research aims to explore the influence and mechanism of Dextran Sulfate (DS) affecting EMT of human gastric cancer. In the present study, we found that DS can enter into the cytoplasm and function in it. Inhibition of HIF-1α or DS significantly inhibit the migration and invasion of human gastric cancer cells, and decrease the mRNA and protein expressions of HIF-1α, matrix metalloproteinase-2 (MMP-2), transforming growth factor-β (TGF-β), Twist and N-cadherin (N-cad), rise E-cadherin (E-cad) expression, DS with HIF-1α knockdown has a stronger effect. *In vivo* studies indicated that compared with using DS or HIF-1α knockdown alone, DS with HIF-1α knockdown can better suppress the volume and number of metastatic tumors, and reduce the mRNA and protein expressions of HIF-1α, MMP-2, TGF-β, Twist and N-cad in metastatic tumor tissues of nude mice. We further demonstrated that the expression of HIF-1α, MMP-2, TGF-β , Twist and N-cad were higher in well and poorly differentiated gastric cancer than paracancerous tissue, and poorly differentiated gastric cancer were even higher, while E-cad expression was opposite. Taken together, this study shows that DS can interfere the expression of HIF-1α, thereby inhibiting TGF-β-mediated EMT of gastric cancer cells, and demonstrated a promising application of DS in gastric cancer therapy.

## Introduction

The peritoneal implant metastasis is one of the main pathways for gastric cancer metastasis and also the main cause for high mortality 1. Currently the main treatment relies on drugs which mean the researches of intraperitoneal implantation and metastasis of gastric cancer have irreplaceable significance for gastric cancer treatment 2. Dextran sulfate (DS) is a macromolecular dextran, the previous researches have shown that DS has an inhibitory effect on the EMT of gastric cancer, but its mechanism hasn't been recognized yet. Hypoxia is one of basic characteristics of physical microenvironment of solid tumors 3. In hypoxia microenvironment, tumor cells highly express hypoxia inducible factor-1 α (HIF-1α) 4. The expression of HIF-1α promotes tumor can be raised by promoting EMT in tumor cells 5-6. Transforming growth factor-β (TGF-β) plays an important role in the process of EMT, metastasis and invasion of cancer cells 7-8. Key factors such as HIF-1α, metalloproteinase (MMP) and Twist (a highly conserved transcription factor) cross-link with EMT signal pathway in cells to regulate the important markers of EMT (E-cadherin, N-cadherin) and lead to induce EMT in tumor cells and boost tumor growth and metastasis 9-13. This research aims to explore the mechanism of DS affecting EMT in the process of human gastric cancer metastasis by inhibiting HIF-1α through *in vitro* experiments, animal experiments and immunohistochemical. These results will provide theoretical basis for DS treatment of abdominal cancer metastasis of gastric cancer.

## Materials and methods

### Cells and transfection

The human gastric adenocarcinoma cell line HGC-27 was purchased from Cell Bank of the Chinese Academy of Science (Shanghai, China). AGS cell line was obtained as a gift from Tangshan Yanyi Biotechnology Co., Ltd (Tangshan, China). HIF-1α-shRNA (shHIF-1α) and NC-shRNA (shNC) lentiviral vectors were purchased from Hanheng Biotechnology Co., Ltd (Shanghai, China). The HIF-1α-shRNA and NC-shRNA oligonucleotide coding sequence is shows in Table 1.

### Drugs

Dextran sulfate (DS), with a molecular weight of 500,000, was purchased from Sigma USA. DS was dissolved in phosphate buffered saline (PBS) or physiological saline and then sterilized using a 0.22 μm filter. The final concentration is 0.3% for cell culture or animal experiments.

### Reagent

Primary antibody HIF-1α (20960-1-AP), MMP-2 (10373-2-AP), Twist (25465-1-AP), E-cadherin (20874-1-AP), N-cadherin (22018-1-AP) were purchased from ProteinTech Group, Inc(Wuhan, China),TGF-βwas purchased from Abcam (ab27969; Abcam, Cambridge, MA, USA), Goat anti-rabbit IgG (ZB-2301) and rabbit two-step detection kit (PV-9001) were purchased from Beijing Zhongshan Jinqiao Biotechnology Co., Ltd (Beijing, China). Total RNA Kit II RNA extraction kit (R6934-01), reverse transcription kit was purchased from OMEGA Biotechnology Co., Ltd. Primary fetal bovine serum was purchased from Bioind (04-001-1ACS). The whole protein extraction kit (KGP250) was purchased from KGI Biotechnology Co., Ltd (Jiangsu, China).

### Animal

BALB/c Nude nude mice were purchased from Beijing Weitong Lihua Experimental Animal Technology Co., Ltd. (Animal license number SCXX (Jing) 2006-0009), 5-6 weeks old, male, weighing 18-22 g. The animals were kept under the SPF conditions of the Experimental Animal Center of Ningxia Medical University, and free access to sterile food and water.

### Human gastric cancer tissue specimens

Total of 36 gastric cancer patients underwent surgery at Ningxia People's Hospital (Yinchuan, China) from October 2018 to October 2019 were recruited. None of the patients received pre-operative chemotherapy or radiation therapy and were diagnosed with gastric cancer by two pathologists according to the ESMO-ESSO-ESTRO clinical practice guidelines. The informed consent of all individuals has been obtained for this study. The collected gastric cancer and adjacent tissues were used for immunohistochemistry experiments.

### Cell culture and transfection

Human gastric cancer cells (HGC-27, AGS) were cultured in a sterile constant temperature incubator at 37 °C, 5% CO2, respectively. HGC-27 cells were cultured in 1640 medium containing 10% FBS, AGS cells were cultured in F12 complete medium containing 10% FBS. The cells in logarithmic growth phase were cultured in a 24-well plate to about 70% confluence, then replaced with serum-free culture medium and divided into control group, shNC group, shHIF-1α group, DS group, DS + shHIF-1α group, which respectively added PBS, shNC lentiviral vectors and shHIF -1α lentiviral vectors, DS and DS with shHIF -1α lentiviral vectors. The fresh medium was changed after 6h and the culture was continued for 24h for subsequent experiments.

### Transwell assay

Cells were seeded in the upper chamber of the transwell insert in serum-free medium after being serum-starved overnight. For migration assay, the lower chamber was filled with RPMI-1640 culture medium containing 10% FBS as chemoattractant. For invasion assay, cells were seeded in the upper chamber of the inserts that were precoated with matrigel. After incubation for 24 h at 37°C, the cells remaining in the upper chamber were wiped away with a cotton swab. The cells adhering to the lower surface of the filter were fixed with 4% polyoxymethylene and stained with 0.1% crystal violet for 30 min. The images of invaded or migrated cells were captured and number of cells was counted under the inverted microscope.

### Q-PCR

The total mRNA of the experimental tissues and cells was extracted according to the manufacturer's protocols provided by OMEGA's Total mRNA Kit II kit, and reverse transcription kit (OMEGA) was used to reverse transcription to generate cDNA. The PCR amplification system contains 25 uL, including ddH20 8.5 uL, upstream and downstream primers 1uL, cDNA 2 uL, and SYBR Green Mix Tag 12.5 uL. Reaction conditions: 40 cycles of 94°C 30 s, 94°C 5 s, and 56°C 30 s, then 65°C 5 s and 95°C 5 min. The primer sequences designed are listed in Table 1. All genes expression was normalized to GADPH, relative mRNA expression of target factor was calculated using 2^-ΔΔCt^ method.

### Western-blot

Total proteins were extracted from tissue and treated cells using a protein extraction kit (KGP2100; Nanjing KeyGen Biotech Co., Ltd., Nanjing, China), then centrifuged and extracted the supernatant. Protein concentration was measured using the BCA Protein Quantification Kit (Nanjing KeyGen Biotech Co., Ltd.). Equal quality of protein (50 μg) were separated by 8-10% SDS-PAGE and transferred to polyvinylidene difluoride membranes, then blocked in 5% non-fat milk for 30 min, and then incubated with the corresponding primary antibodies at 4°C overnight. Subsequently, the membrane was incubated with horseradish peroxidase-conjugated secondary antibodies at room temperature for 1 h. An enhanced chemiluminescence reagent (ECL kit, KGP1121; Nanjing KeyGen Biotech Co., Ltd.) was applied as a chromogenic substrate for 1 min, then visualized with an Amersham Imager 600 instrument (GE Healthcare Life Sciences). The purpose/internal reference is used to indicate the expression level of the corresponding protein. Grayscale analysis was performed with ImageJ software.

### Immunohistochemistry

Immunohistochemistry (IHC) was performed as previously described 12. Briefly, the 4μm tissue sections were incubated with corresponding primary antibodies as follows: HIF-1α (1:700), TGF-β (1:150), Twist (1:200), MMP-2 (1:1000), E-cadherin (1:300), N-cadherin (1:700), negative control uses PBS instead of primary antibody. Then incubated with rabbit two-step detection kit, finally stained with 3,3-diaminobenzidine and hematoxylin. Figureures analysis uses the same method as immunocytochemistry.

### Nude mice mode of gastric cancer abdominal celiac metastasis

Ninety BALB/c Nude mice were randomly divided into Control group, shNC group, shHIF-1α group, DS group, DS+shHIF-1α group. The suspension of HGC-27 cells and transfected shNC and shHIF-1α HGC-27 cells was adjusted to a concentration of 1×10^7^ cells/ml, and 0.2 ml of cell suspension was injected to abdominal cavity of nude mice. The whole process of the experiment is strictly in accordance with aseptic operation. On the fourteenth day after surgery, nude mice were sacrificed by cervical dislocation. Then observe the number, size, and color of tumor nodules in the abdominal cavity, especially on the omentum, and collect two copies of the omentum tissue implanted with tumor nodules. One tissue was fixed with 4% paraformaldehyde fixation solution, the other was placed in a cryopreserved test tube pre-sterilized and treated with DEPC water, and frozen at -80°C for subsequent experiments. The animals were kept under the SPF conditions of the Experimental Animal Center of Ningxia Medical University, and free access to sterile food and water. All experiments were approved by the ethics committee of Ningxia Medical University, and took place at Ningxia Medical University.

### Statistical analysis

All experiments were repeated at least three times in duplicate. SPSS 22.0 software was used to analyze all the statistical dates. Data are presented as the mean ± SD. The two-sample means were compared using the t-test of the two-sample means. The two-factor correlation analysis was measured Pearson correlation analysis. One‐way ANOVA was used as mean comparison method among different groups. P‐values <0.5 were considered as a statistical significance.

## Results

### *In Vitro* experiments

#### Fluorescence Microscopy

**Figure 1:**
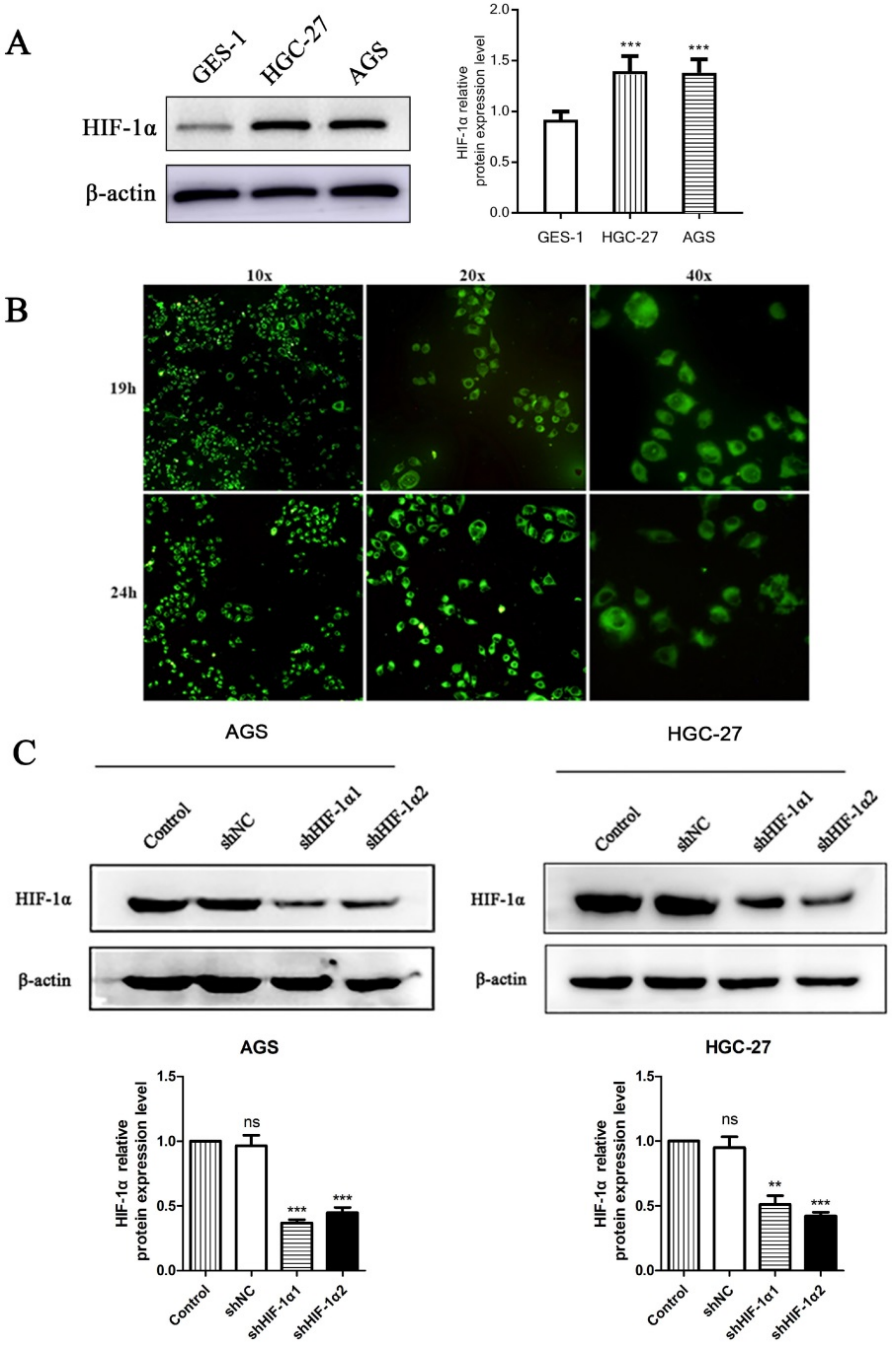
** (A)** Western blot shows the HIF-1α expression level in gastric cancer cells and normal gastric epithelial cell (*P<0.001 compared with GES-1 group). **(B)** Fluorescence microscopy observed that DS entered the cytoplasm of human gastric cancer cells (HGC-27) 19 h and 24 h after DS intervention. **(C)** Western blot showed that the HIF-1α was successfully knockdown in human gastric cancer cells (ns, **P<0.01, ***P<0.001 compared with Control group).

We used western blot observed the protein expression level of HIF-1α in two different gastric cancer cell lines and a nomal gestric epithelial cell (GES-1). The results showed that the expression level of HIF-1α in gastric cancer cell lines were higher than that of GES-1 (P<0.001) (Figure 1A). Use DS with fluorescent labels to treat cells and observe the distribution of DS after entering the cells. HGC-27 cells showed a weak fluorescence after DS treated for 8 h. After 19 h of intervention, the intracellular fluorescence intensity was strong, and the drug was mainly distributed in the cytoplasm after entering the cell. The drug was evenly distributed in the cytoplasm after 24 h of intervention. The complete cell structure and fluorescence distribution could not be observed at 32 h of intervention (Figure 1B). Use shRNA to knockdown HIF-1α in gastric cancer cells (AGS, HGC-27), Western blot results showed that the expression of HIF-1α in human gastric cancer cells was successfully knocked down (P<0.01, P<0.001) (Figure 1C).

#### Transwell migration and invasion

**Figure 2:**
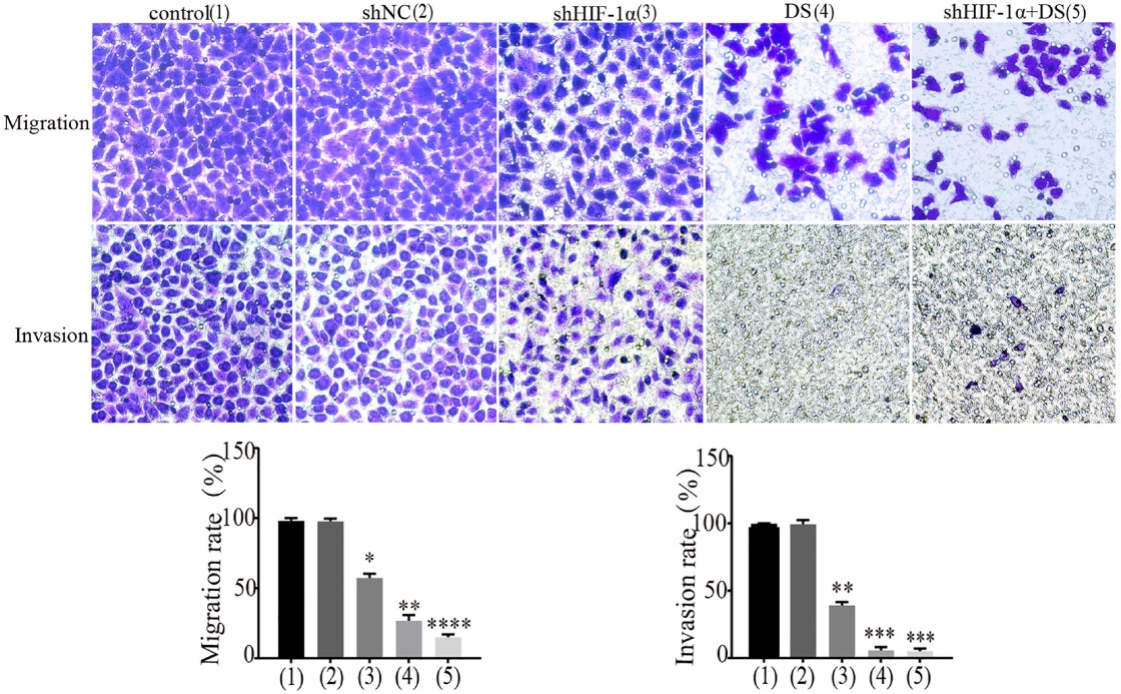
Transwell experiment detect the ability of cell invasion and metastasis in each group (**P<0.01, ***P<0.001, ****P<0.0001 compared with Control or shNC group).

We performed transwell migration and wound healing assays of human gastric cancer cells (AGS) to demonstrate the role of HIF-1α and DS play in gastric cancer cells metastasis. The results showed that the migration and invasion of shHIF-1α group, DS group and DS + shHIF-1α group were significantly weaker than that of the control group (P<0.05, P<0.01,P<0.01); the migration and invasion of cells in DS + shHIF-1α group were significantly weaker than that of shHIF-1α group(P<0.01) (Figure 2).

#### QRT-PCR detect the mRNA expression of HIF-1α, MMP-2, TGF-β, Twist, N-cad, E-cad in each group of cells

**Figure 3:**
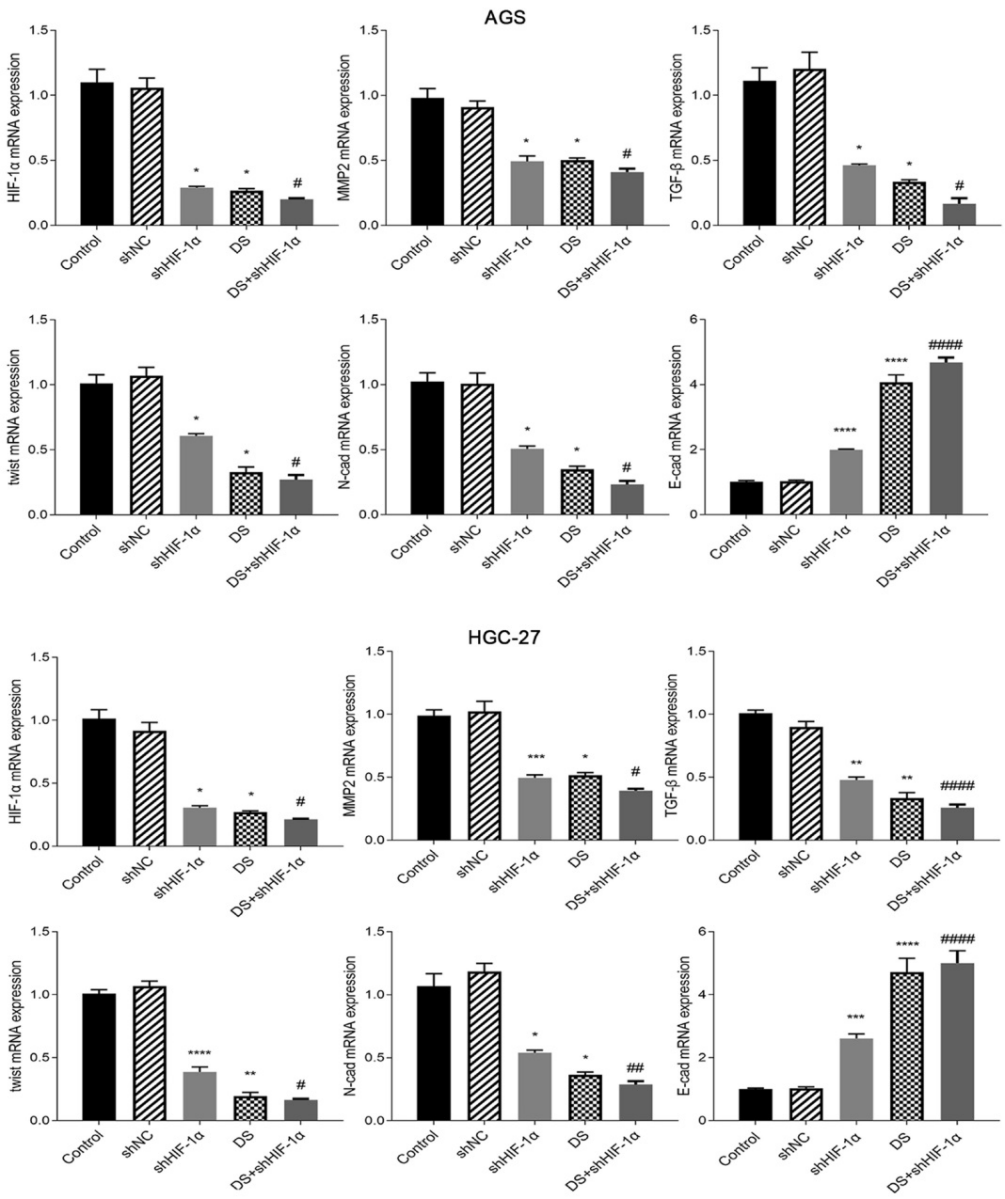
QRT-PCR detect the mRNA expression of HIF-1α, MMP-2, TGF-β, Twist, N-cad, E-cad in each group of cells (*p<0.05, **p<0.01, ***p<0.001, ****p<0.0001 compared with Control group; #p<0.05, ##p<0.01, ###p<0.001, ###p<0.0001 compared with shHIF-1α group).

Human gastric cancer cell (AGS) experimental results showed that the mRNA expressions of HIF-1α, MMP-2, TGF-β, Twist and N-cad in shHIF-1α group, DS group and DS+shHIF-1α group were significantly lower than those in control group, E-cad mRNA expression was significantly higher than the control group (p<0.05 or p<0.01). Compared with the shHIF-1α group with the DS + shHIF-1α group, the mRNA expression differences of HIF-1α, MMP-2, TGF-β, Twist, N-cad, and E-cad were statistically significant. In the same experiment, human gastric cancer cells (HGC-27) obtained the same experimental results (Figure 3).

#### Western blot detect the protein expression of HIF-1α, TGF-β, MMP-2, Twist, N-cad, E-cad in each group of cells

**Figure 4:**
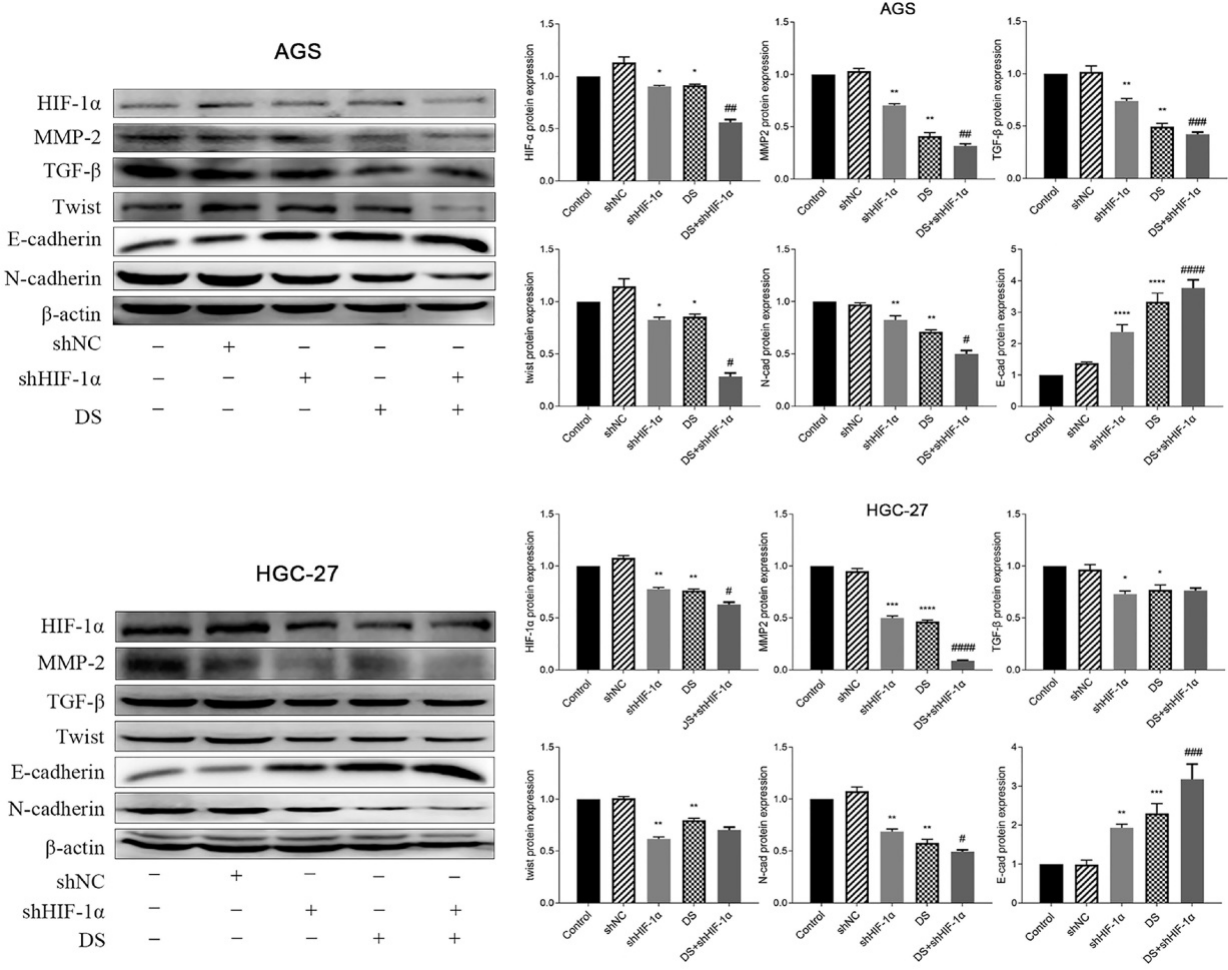
Western blot detect the protein expression of HIF-1α, TGF-β, MMP-2, Twist, N-cad, E-cad in each group of cells (*p<0.05, **p<0.01, ***p<0.001, ****p<0.0001 compared with Control group; #p<0.05, ##p<0.01, ###p<0.001, ###p<0.0001 compared with shHIF-1α group).

The western blot results showed that the protein expressions of HIF-1α, MMP-2, TGF-β, Twist and N-cad in gastric cancer cell (AGS) in the shHIF-1α group, DS group and DS+shHIF-1α group were significantly lower than those in the control group. The protein expression of E-cad was significantly higher than that of the control group (p<0.05, p<0.01). The DS+shHIF-1α group had a more significant effect than the shHIF-1α group (p<0.05, p<0.01). Human gastric cancer cells (HGC-27) obtained the same experimental results, the results showed that the expression of HIF-1α, MMP-2, TGF-β, Twist and N-cad in shHIF-1α group, DS group and DS+shHIF-1α group was significantly reduced, while the protein expression of E-cad was significantly increased (p<0.05, p<0.01). Compared the protein expression differences of DS+shHIF-1α group with shHIF-1α group, the protein expression of HIF-1α, MMP-2, N-cad, and E-cad were statistically significant (p<0.05, p<0.01), and there was no significant difference in protein expression between TGF-β and Twist (Figure 4).

### Animal experiment

#### Abdominal cavity metastasis in nude mice

**Figure 5:**
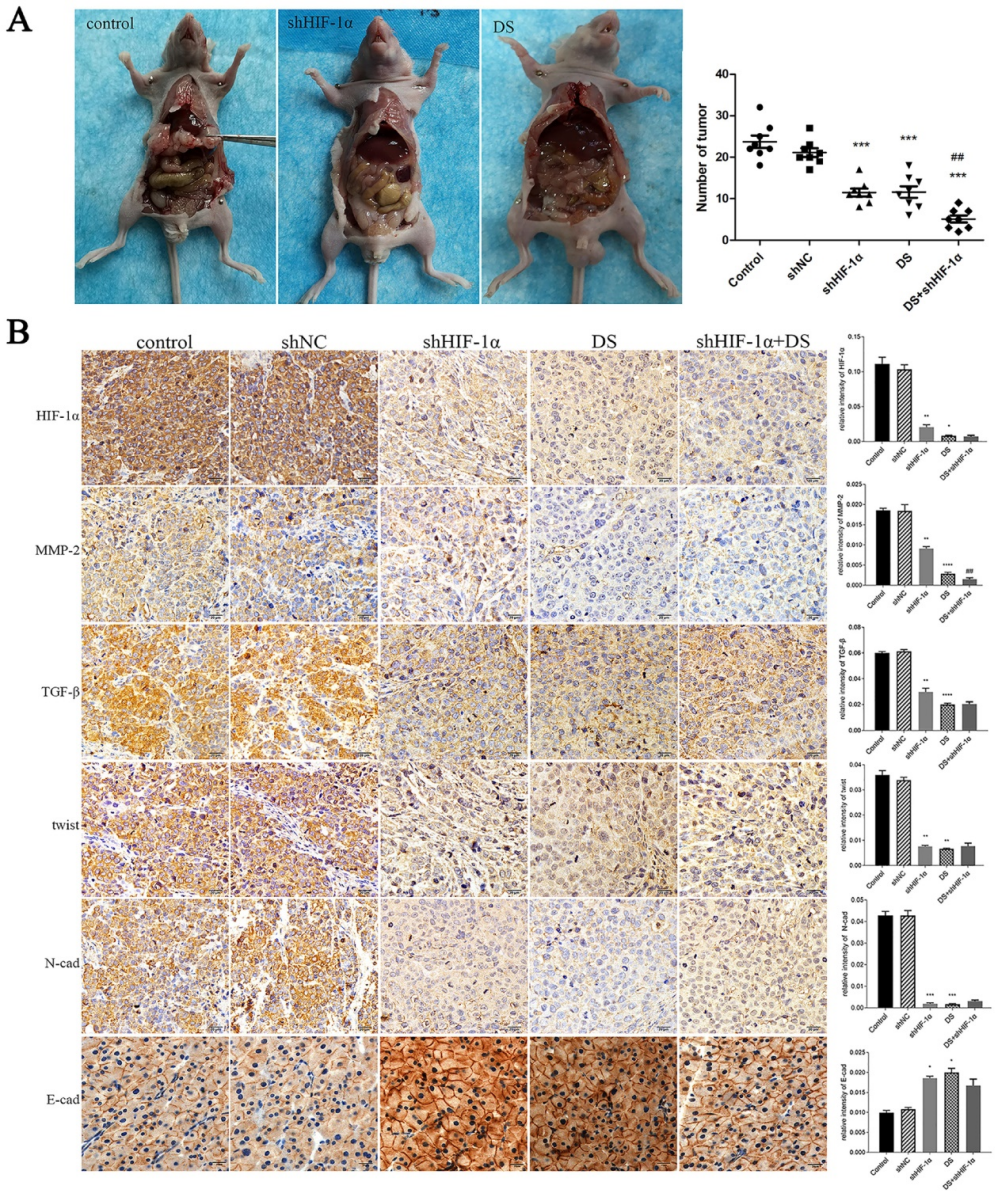
**(A)** Observation of the abdominal cavity metastasis in nude mice in each group. **(B)** Immunohistochemistry detect the expression of HIF-1α, MMP-2, TGF-β, Twist, N-cad, and E-cad in metastatic tumor tissues of nude mice (*p<0.05, **p<0.01, ***p<0.001, ****p<0.0001 compared with Control group; ##p<0.01 compared with shHIF-1α group).

In the shHIF-1α group, DS group, and DS+shHIF-1α group, the number of metastatic tumors in the abdominal cavity of nude mice is less than that of the control group (p<0.01, p<0.001), and the volume of the tumor nodules is smaller than that of the control group. The tumor nodules are porcelain white and hard (Figure 5A).

#### Immunohistochemistry detect the expression of HIF-1α, MMP-2, TGF-β, Twist, N-cad, and E-cad in metastatic tumor tissues of nude mice

The expressions of HIF-1α, MMP-2, TGF-β, Twist and N-cad in the shHIF-1α group, DS group and DS + shHIF-1α group were significantly lower than those in the control group, and the expressions of E-cad were significantly higher than those in the control group (p<0.05 or p<0.01). Compared with the group of DS+shHIF-1α and shHIF-1α, there was no significant difference in the expression of other factors except MMP-2 (p<0.01) (Figure 5B).

#### QRT-PCR detect mRNA expression of HIF-1α, MMP-2, TGF-β, Twist, N-cad, E-cad in metastatic tumor tissues of nude mice

**Figure 6:**
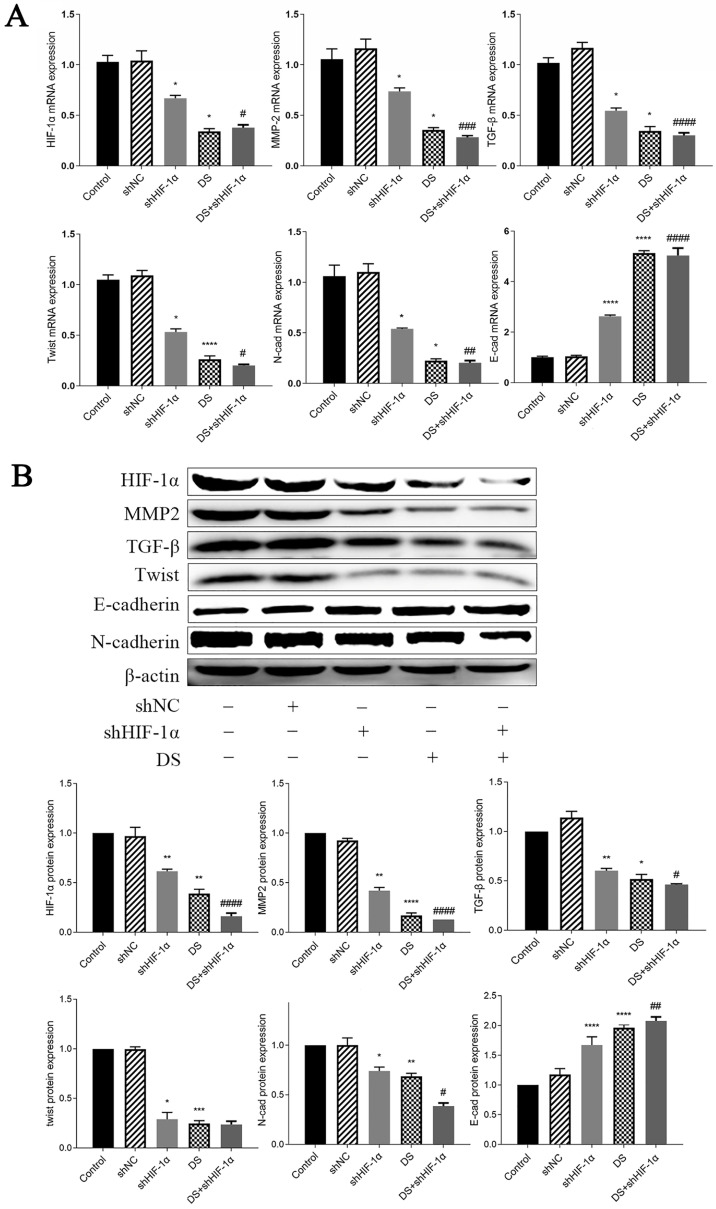
**(A)** QRT-PCR detect mRNA expression of HIF-1α, MMP-2, TGF-β, Twist, N-cad, E-cad in metastatic tumor tissues of nude mice (*p<0.05, **p<0.01, ***p<0.001, ****p<0.0001 compared with Control group; #p<0.05, ##p<0.01, ###p<0,0001 compared with shHIF-1α group). **(B)** Western blot detect the expression of HIF-1α, MMP-2, TGF-β, Twist, N-cad, and E-cad proteins in metastatic tumor tissues of nude mice (*p<0.05, **p<0.01, ***p<0.001, ****p<0.0001 compared with Control group; #p<0.05, ##p<0.01, ###p<0.0001 compared with shHIF-1α group).

The mRNA expression of HIF-1α, MMP-2, TGF-β, Twist, and N-cad in the shHIF-1α group, DS group, and DS+shHIF-1α group was significantly lower than that of the control group, while the mRNA expression of E-cad was significantly higher (p <0.05, p<0.01). Compared the mRNA expression in shHIF-1α group with DS+shHIF-1α group, the mRNA expression of HIF-1α, MMP-2, TGF-β, Twist, N-cad, and E-cad were statistically significant (p<0.05, p<0.01) (Figure 6A).

#### Western blot detect the expression of HIF-1α, MMP-2, TGF-β, Twist, N-cad, and E-cad proteins in metastatic tumor tissues of nude mice

The expressions of HIF-1α, MMP-2, TGF-β, Twist and N-cad in shHIF-1α group, DS group and DS+shHIF-1α group were significantly lower than those in the control group, and the protein expression of E-cad were significantly higher than those in the control group (p<0.05, p<0.01). The difference in protein expression of HIF-1α, MMP-2, TGF-β, N-cad, and E-cad between the DS+shHIF-1α group and the shHIF-1α group was statistically significant (p<0.05, p<0.01); However, there was no significant difference in the protein level of Twist between the DS+shHIF-1α group and the shHIF-1α group (Figure 6B).

### Human gastric cancer tissue research

#### Immunohistochemistry detect the expression of HIF-1α, MMP-2, TGF-β, Twist, N-cad, and E-cad in gastric cancer tissues

**Figure 7:**
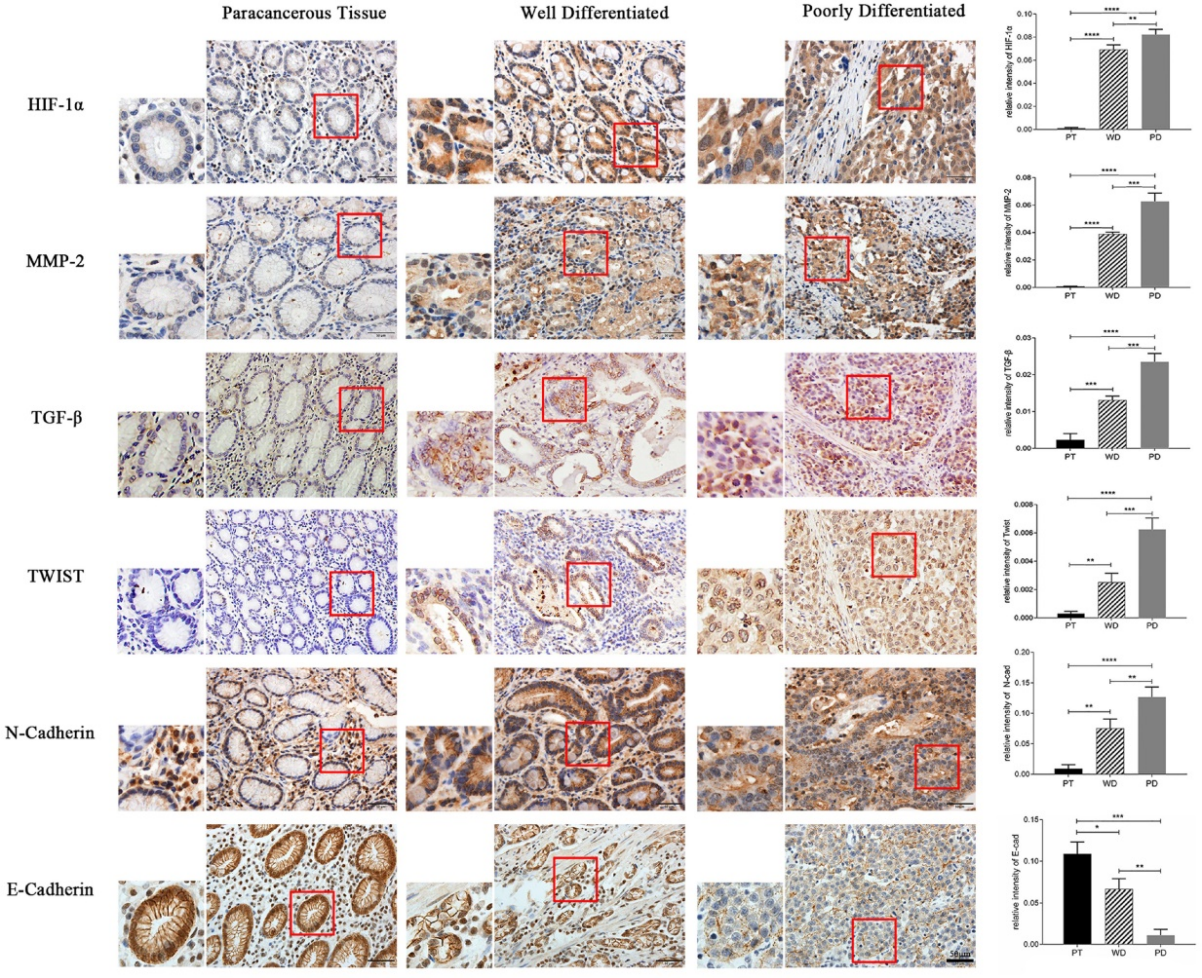
Immunohistochemistry detect the expression of HIF-1α, MMP-2, TGF-β, Twist, N-cad, and E-cad in gastric cancer tissues (*p<0.05, **p<0.01, ***p<0.001, ****p<0.0001).

The results of immunohistochemical staining showed that the expression of HIF-1α, MMP-2, TGF-β, Twist, and N-cad in both highly differentiated and poorly differentiated cancer tissues was significantly higher than that in normal adjacent tissues (p<0.01, p<0.001), while E-cad expression was opposite (p<0.05, p<0.01). Moreover, the expression of HIF-1α, MMP-2, TGF-β, Twist, and N-cad was significantly higher in poorly differentiated than in highly differentiated tissue, and the expression of E-cad in well-differentiated was significantly higher than in poorly differentiated (p<0.05, p<0.01) (Figure 7).

## Discussion

Peritoneal implant metastasis is one of the main pathways for gastric cancer metastasis. Gastric cancer metastasis is a complex process. EMT is closely related to metastasis of gastric cancer cells. Our research group has carried out the study on the treatment of Intraperitoneal implant metastasis gastric cancer with DS. The biological characteristics of DS are related to its molecular weight. Micro molecule DS (below 1000Da) has effect of inhibiting virus 14. DS with molecular weight from 1000Da to 40000Da can boost the growth of ovarian stem cells in Chinese hamsters 15, and it also inhibit the migration of pancreatic ductal cancer and breast cancer by inhibiting the expression of hyaluronidase 16-17. Our research group conducted a study on the treatment of gastric cancer peritoneal implantation metastasis by using macromolecule DS (500,000 Da) which has the characteristics of slow absorption and long action time in the abdominal cavity. Studies have shown that DS has an inhibitory effect on EMT of gastric cancer 12-13, but the pathway and mechanism are still unclear. In this study, after co-cultured gastric cancer cells with DS for 18 hours, it entered into gastric cancer cells which indicating that DS may enter into gastric cancer cells and cause function of cells changes.

Multiple signaling factors in tumor tissues interact with each other and mediate the formation of EMT and initiate tumor metastasis by inducing changes in morphology and function of tumor cell 18. Some studies on gastric cancer showed that the expression of HIF-1α in patients with peritoneal metastasis was obviously higher than in whom without it, which may be a predictor of the therapeutic effect of gastric cancer patients^19^.

In hypoxia microenvironment, tumor cells highly expressed hypoxic inducible factor -1α (HIF-1α) 20. EMT in tumor cells can be promoted by increasing expression of HIF-1α which result to tumor's growth and metastasis 21. HIF-1α inhibits the expression of E-cad, promotes the expression of N-cad, and induces EMT in tumor cells by regulating the transcription factors Twist (a highly conserved transcription factor) and snail if tumor cells are hypoxic 22. Transforming growth factor-β (TGF-β) and matrix metalloproteinases (MMPs) also play an important role in the process of EMT and both are closely related to HIF-1α 23. HIF-1α can up regulate the expression levels of TGF-β and MMPs, affect the crosstalk of signal pathway factors in tumor cells and regulate important marker factors (E-cad, N-cad) of EMT. In this case, HIF-1α induces EMT in tumor cells and promotes tumor growth and metastasis 9-11.

The experiment results of transwell migration and invasion of human gastric cancer cells (AGS) showed that the migration and invasion of shHIF-1α group, DS group and DS + shHIF-1α group were significantly weaker than that of the control group; the migration and invasion of cells in DS + shHIF-1α group were significantly weaker than that of shHIF-1α group, which means HIF-1α can promote the migration and invasion of human gastric cancer cells. Meanwhile, adding DS has the same effect as knocking down HIF-1α which means DS can reduce the migration and invasion ability of human gastric cancer cells by effecting HIF-1α. In addition to HIF-1α knockdown, DS has a superimposed effect, which has a more obvious inhibitory effect on the migration and invasion of human gastric cancer cells, further indicating that DS can inhibit the migration and invasion of human gastric cancer cells through HIF-1α.

The results of this study *in vitro* showed that the mRNA and protein expression of HIF-1α, MMP-2, TGF-β, Twist, N-cad was significantly lower than which of the control group after knocking down HIF-1α. The mRNA and protein expression of E-cad was significantly higher than that of the control group. The differentiation of above conclusion has statistical significance which means HIF-1α can promote EMT at mRNA and protein level. Adding DS has the same effect on EMT as the knockdown of HIF-1α which indicating that DS can inhibit EMT of gastric cancer cells by acting HIF-1α. DS addition knocking down the HIF-1α produced a superposition effect, which strengthens the promotion of EMT, and has a more obvious inhibition effect. Furthermore, DS can inhibit the development of EMT in gastric cancer cells by affecting the expression of important EMT markers (E-cad and N-cad) at mRNA and protein level by acting HIF-1α. The results are the same in well differentiated human gastric cancer cells (AGS) and poorly differentiated human gastric cancer cells (HGC-27), which suggesting that DS could inhibit EMT of gastric cancer cells at mRNA and protein level through HIF-1α in human gastric cancer cells with different degrees of differentiation.

We did further experiments to verify whether DS can inhibit EMT and peritoneal metastasis of gastric cancer cells through HIF-1 *in vivo*. The results showed that the number of metastatic tumors and the volume of tumor nodules in the abdominal cavity of nude mice decreased significantly after the knockdown of HIF-1α or treatment with DS, which indicated that indicating DS can inhibit peritoneal implantation and metastasis of gastric cancer as well as the knockdown of HIF-1α. The results of HIF-1α, MMP-2, TGF-β, Twist, N-cad and E-cad by extracting mRNA and protein from peritoneal implant metastasis are consistent with those of *in vitro* which further verified the promoting effect of HIF-1α on EMT process, and the knockdown of HIF-1α can inhibit the peritoneal implant metastasis of gastric cancer. DS can inhibit peritoneal implant metastasis of gastric cancer by affecting EMT of gastric cancer cells through the action of HIF-1α.

Hypoxia is one of the basic characteristics of physical microenvironment of solid tumors. In the process of tumor formation, if oxygen demand of local tumor tissue is not satisfied, or the immature blood vessel collapses due to the increase of interstitial pressure, the local microenvironment will expose to hypoxia situation. It can be found in most solid tumors that EMT occurs in the tumor central anoxic region of cells. For further improvement of expression of the above factors (HIF-1α, MMP-2, TGF-β, Twist, N-cad and E-cad) affecting EMT in human gastric cancer, in this study, immunohistochemical staining was carried out in poorly differentiated and highly differentiated gastric adenocarcinoma and its adjacent tissues. The results showed that the protein expression of HIF-1α, MMP-2, TGF-β, Twist and N-cad in cancer tissues was significantly higher than in adjacent tissues, and the expression of HIF-1α, MMP-2, TGF-β, Twist, N-cad has increased gradually in adjacent tissues, highly differentiated adenocarcinoma and poorly differentiated adenocarcinoma. Oppositely, the protein expression of E-cad decreased gradually in adjacent tissues, highly differentiated adenocarcinoma and poorly differentiated adenocarcinoma. The results showed that EMT makers is obvious in gastric cancer than in adjacent tissues, which means the lower the differentiation level, the EMT occurs are easier to be observed, and these factors have great influence on the development of tumor.

In summary, after knocking down of HIF-1α, the gene and protein expression levels of N-cad and its related factors MMP-2, TGF-β, Twist have been decreased significantly, while the gene and protein expression levels of E-cad increased significantly. It rises a conclusion of EMT can be inhibited, also indicating HIF-1α can promote gene and protein levels of gastric cancer cells to EMT. The effect of DS on EMT is the same as which on HIF-1α knockdown, and adding DS after HIF-1α knockdown has a superposition effect. Thus, the promotion effect is enhanced and the inhibitory effect is highly observable. DS can enter into gastric cancer cells indicating that DS may affect the biological function of gastric cancer cells. DS can interfere the expression of HIF-1α, thereby inhibiting TGF-β-mediated EMT of gastric cancer cells, and demonstrated a promising application of DS in gastric cancer therapy.

## Figures and Tables

**Table 1:** The sequences of shRNA and primers employed in study.

Primer Name	Primer Sequence
shRNA-HIF-1α Top strand	GATCCGCACCTATGACCTGCTTGGTGCTGATTTCAAGAGAATCAGCACCAAGCAGGTCATAGGTGTTTTTTG
shRNA-HIF-1α Bottom strand	AATTCAAAAAACACCTATGACCTGCTTGGTGCTGATTCTCTTGAAATCAGCACCAAGCAGGTCATAGGTGCG
shNC Top strand	GATCCGTTCTCCGAACGTGTCACGTAATTCAAGAGATTACGTGACACGTTCGGAGAATTTTTTC
shNC Bottom strand	AATTGAAAAAATTCTCCGAACGTGTCACGTAATCTCTTGAAT-TACGTGACACGTTCGGAGAACG
HIF-1α Forward	GAAAGCGCAAGTCTTCAAAG
HIF-1α Reverse	TGGGTAGGAGATGGAGATGC
TGF-β Forward	GACACCAACTATTGCTTCAG
TGF-β Reverse	CAGGCTCCAAATGTAGGG
Twist Forward	AATTGGGATGCATTCGAGTCTGTAA
Twist Reverse	TTCTGTCCGATGTCACTGCTGTC
E-cadherin Forward	TACACTGCCCAGGAGCCAGA
E-cadherin Reverse	TGGCACCAGTGTCCGGATTA
N-cadherin Forward	ACCTGAACGACTGGGGGCCA
N-cadherin Reverse	TGCCAAAGCCTCCAGCAAGCA
GADPH Forward	CAAGGTCATCCATGACAACTTTG
GADPH Reverse	GTCCACCACCCTGTTGCTGTAG
